# Wheat Bran Polyphenols Ameliorate DSS-Induced Ulcerative Colitis in Mice by Suppressing MAPK/NF-κB Inflammasome Pathways and Regulating Intestinal Microbiota

**DOI:** 10.3390/foods13020225

**Published:** 2024-01-10

**Authors:** Xusheng Wen, Han Peng, Hua Zhang, Yangzheng He, Fanghua Guo, Xin Bi, Jiahua Liu, Yong Sun

**Affiliations:** 1 State Key Laboratory of Food Science and Resources, Nanchang University, Nanchang 330047, China; 407900210013@email.ncu.edu.cn (X.W.); 357900220006@ncu.edu.cn (Y.H.); gfh1376234247@outlook.com (F.G.); bixin@email.ncu.edu.cn (X.B.); 417900210017@email.ncu.edu.cn (J.L.); 2 Department of Food Science and Technology, University of California, Davis, 1 Shields Ave., Davis, CA 95616, USA; hanp@mun.ca; 3 School of Pharmacy, Jiangxi University of Traditional Chinese Medicine, Nanchang 330004, China; 20091022@jxutcm.edu.cn

**Keywords:** wheat bran polyphenols, ulcerative colitis, inflammation, intestinal barrier function, gut microbiota

## Abstract

Wheat bran (WB) is the primary by-product of wheat processing and contains a high concentration of bioactive substances such as polyphenols. This study analyzed the qualitative and quantitative components of polyphenols in wheat bran and their effects on ulcerative colitis (UC) using the dextran sulfate sodium (DSS)-induced colitis model in mice. The potential mechanism of wheat bran polyphenols (WBP) was also examined. Our findings indicate that the main polyphenol constituents of WBP were phenolic acids, including vanillic acid, ferulic acid, caffeic acid, gallic acid, and protocatechuic acid. Furthermore, WBP exerted remarkable protective effects against experimental colitis. This was achieved by reducing the severity of colitis and improving colon morphology. Additionally, WBP suppressed colonic inflammation via upregulation of the anti-inflammatory cytokine IL-10 and downregulation of pro-inflammatory cytokines (TNF-α, IL-6, IL-1β) in colon tissues. Mechanistically, WBP ameliorated DSS-induced colitis in mice by inhibiting activation of the MAPK/NF-κB pathway. In addition, microbiome analysis results suggested that WBP modulated the alteration of gut microbiota caused by DSS, with an enhancement in the ratio of *Firmicutes/Bacteroidetes* and adjustments in the number of *Helicobacter*, *Escherichia-Shigella*, *Akkermansia*, *Lactobacillus*, *Lachnospiraceae_NK4A136_group* at the genus level. To conclude, the findings showed that WBP has excellent prospects in reducing colonic inflammation in UC mice.

## 1. Introduction

Ulcerative colitis (UC) as a kind of inflammatory bowel disease (IBD) is characterized by clinical symptoms such as diarrhea, blood in the stool, weight loss, and colonic ulcers [1,2]. If left untreated and uncontrolled, UC can progress into colon cancer [3,4]. The pathogenesis of UC is intricate, and although its mechanisms of onset and development have not been fully elucidated, studies have suggested that it is closely linked to the immune system and intestinal microbes [5]. Currently, the primary treatments for UC involve the use of anti-inflammatory medications and immunosuppressants [6]. However, these drugs often come with severe side effects and adverse reactions when used long term [7]. Further studies have demonstrated that certain natural compounds, such as polyphenols, flavonoids, and dietary fiber, have the potential to prevent and alleviate UC symptoms [8,9].

Polyphenols are a group of phytochemicals that are abundant in various plants commonly found in diets. They have been widely studied for their diverse bioactive effects, including antioxidation, anti-inflammation, and bacteriostasis, which contribute to human health [10]. Relevant data support the notion that a diet rich in polyphenols can both prevent and alleviate the symptoms of IBD [11,12]. Interestingly, the majority of polyphenols, accounting for approximately 90% to 95%, are not assimilated by the small intestine. Instead, they accumulate in the colon as a substrate for fermentation, are decomposed and metabolized by intestinal microflora, and then absorbed by the human body, thus affecting the onset and progression of IBD [13,14].

The pathogenesis of UC has been linked to dysfunctional intestinal barriers and dysbiosis within the intestinal microbiome [15]. Activation of the mitogen activated protein kinase (MAPK) and nuclear factor kappa-B (NF-κB) inflammatory signaling pathway has been reported as the primary cause of intestinal barrier injury, which can mediate inflammatory responses and promote inflammatory cytokine synthesis [16]. The destruction of the intestinal barrier allows endotoxins and bacteria to invade the intestinal mucosa, leading to intestinal inflammation, disruption of the homeostasis of the intestinal microbiota, and its subsequent exacerbation [4,17]. In recent studies, it has demonstrated that polyphenols possess anti-inflammatory activity, and they can reduce colon inflammation in mice by maintaining the structural integrity of the intestinal barrier and reducing the expression and secretion of inflammatory factors [18]. Additionally, researchers have demonstrated that polyphenols can enhance the balance of gut microorganisms by elevating beneficial bacteria and reducing the presence of harmful bacteria [19]. Ritchie et al. found that a diet enriched with polyphenols from sorghum bran reversed the reduction in gut microbiota diversity and richness induced by DSS, and restored the *Firmicutes*-to-*Bacteroidetes* ratio [20]. Similarly, Zhao et al. reported that the consumption of honey polyphenols could mitigate intestinal inflammation by alleviating oxidative stress damage, downregulating pro-inflammatory cytokines gene expression, and restoring the DSS-affected intestinal microbiota through a reduction in harmful bacteria and an increase in beneficial bacteria [21]. Importantly, some studies have shown the vital function of short-chain fatty acids (SCFAs) in preserving intestinal homeostasis [22]. Therefore, it is worthwhile investigating whether dietary supplementation of polyphenols can improve colitis by regulating the intestinal microbiota’s equilibrium through SCFAs.

Wheat bran is the main by-product of white flour processing and is produced in large quantities globally. It contains a high percentage of nutrients, including carbohydrates (64.51%), protein (15.5%), and minerals (2.92%), as well as various bioactive substances such as polyphenols, flavonoids, and dietary fiber [23,24]. According to Suchowilska et al., the polyphenol extract from wheat bran contains various phenols such as caffeic acid, gallic acid, *p*-coumaric acid, chlorogenic acid, ferulic acid, and others [25]. However, the full physiological functions of WBP have not been discovered. In this study, the primary polyphenol components in WBP were identified by UPLC-ESI-QTOF-MS^2^. Additionally, we examined the in vivo anti-inflammatory capacity of WBP and its potential mechanism of action using the DSS-induced mouse UC model. Meanwhile, the intestinal flora and SCFAs metabolites from the intestinal contents of mice were evaluated. Using all the collected data, we performed a correlation analysis between these indices and the intestinal flora with and without WBP administration. This analysis aims to enhance the understanding of the health benefits of WBP in alleviating UC symptoms in mice.

## 2. Materials and Methods

### 2.1. Materials and Reagents

Wheat bran was supplied by the Canadian International Grains Institute (Winnipeg, MB, Canada). Dextran sodium sulfate (DSS) (36–50 kDa) was obtained from MP Biomedicals (Santa Ana, CA, USA).

### 2.2. Extraction of Polyphenols from Wheat Bran

The wheat bran was dried in an oven at 40 °C and crushed using a grinder (HC-350Y, Wuyi Haina Electric Appliance, Wuyi, China). The resulting powder was then passed through a 100-mesh sieve. Next, the wheat bran powder was weighted to 100 g and mixed with 70% methanol at a ratio of 1:20 of material to liquid. Following ultrasonication for 45 min (50 °C, 800 W), the sample extraction was centrifuged at room temperature (RT) for 10 mins (4000 rpm) to obtain the supernatant; the above operation was repeated three times, and then the supernatant was pooled. The obtained supernatant was frozen and then freeze-dried to produce the wheat bran polyphenol (WBP) extract, which was stored in the refrigerator at −80 °C.

### 2.3. Composition Analysis of Wheat Bran Polyphenols

The main polyphenol component of wheat bran was identified using UPLC-ESI-Q-TOF-MS^2^ (Agilent 6530 Q-TOF LC/MS, Santa Clara, CA, USA). Firstly, the WBP extract was dissolved in a 70% methanol solution at a concentration of 10 mg/mL. After being fully dissolved, the solution was drawn up into a syringe, filtered using a 0.22 μm polyvinylidene fluoride (PVDF) membrane, and injected into a sample vial for further analysis. Subsequently, the substances were separated using an Agilent SB-C18 column (1.8 µm, 2.1 mm × 100 mm). The mobile phases comprised 0.1% formic acid aqueous solution (A) and pure acetonitrile (B). The gradient elution program was set as follows: 0–5 min A, 5–15% B; 5–7 min A, 15% B; 7–12 min A, 15–20% B; 17–22 min A, 20–30% B; 22–24 min A, 30–35% B; 24–30 min A, 35–65% B; 30–33 min A, 65–95% B; 33–36 min A, 95–15% B; and 36–38 min A, 15–5% B. The temperature of the column was maintained at 35 °C, the flow rate was set to 0.3 mL/min, and the injection volume was 5 μL. For the MS conditions, the ESI source was set to negative ionization mode, using full scan mode to obtain mass spectral data across the *m*/*z* range of 50–1000. The ESI conditions were set at the following values: drying gas temperature at 350 °C; drying gas flow at 10 L/min; fragment voltage at 135 eV; and capillary voltage at 4000 V. As for the MS/MS conditions, the collision voltage was set at 15 eV, 25 eV, and 35 eV for analysis.

The quantitative analysis of WBP was carried out by HPLC-VWD (Agilent 1260 HPLC, Santa Clara, USA) and by establishing the corresponding standard curve for phenolic acids. In addition, the liquid chromatographic conditions were based on the method of Zhou et al. with minor modifications [4]. The peaks were then identified by comparing the retention time of compounds between samples and standards. Six reference standards including ferulic acid (0.59 mg), caffeic acid (0.59 mg), *p*-coumaric acid (0.58 mg), protocatechuic acid (1.14 mg), vanillic acid (0.24 mg), and gallic acid (1.13 mg) (Yuanye Bio-Technology, Shanghai, China) were weighed and dissolved in 5 mL of 70% methanol and then diluted with 70% methanol to required concentrations.

### 2.4. Animal Experiments

**Figure 1:**
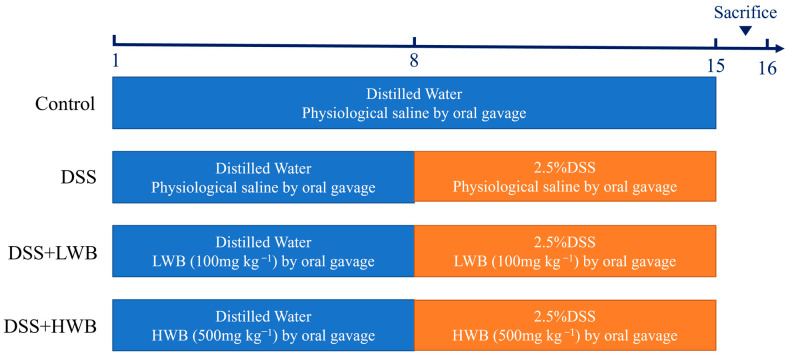
The process diagram of animal experiment.

C57BL/6 J female mice aged 6 to 8 weeks old, were purchased from Hunan Shrek Jingda Experimental Animal Co., Ltd. (Changsha, China), and these animals were housed under controlled environmental conditions with a temperature of 22 °C, a humidity of 55 ± 0.5%, and a 12-h light/dark cycle. The whole experimental process is illustrated in Figure 1. After one week of acclimation, mice were randomly divided into 4 groups (*n* = 10 mice per group), including the control group (CON), model group (MOD), low-dose WBP treatment group (LWB), and high-dose WBP treatment group (HWB). During the experimental period, we measured the weight of the mice every day, and the volume of the gavage was carried out according to the dose of 1 mL/100 g body weight (BW). WBP solution with different concentrations (100 and 500 mg per kg BW) was given to the LWB and HWB group, and normal saline was given to the CON and MOD group. From day one to day seven, the mice had a normal diet and distilled water. On the 8th day, the distilled water of the MOD group and the two WBP groups was replaced with a distilled water solution of 2.5% DSS (*w*/*v*), and the CON group continued to drink distilled water. Each mouse was scored with disease activity index (DAI); the criteria of DAI are shown in Table S1. On the 15th day, mice feces were collected in the metabolic cages and fasted for 12 hours starting at 9 p.m. At 9:00 in the morning on the 16th day, the mice were anesthetized and killed via cervical dissection after taking the whole blood from their eyeballs. Then, the mice were dissected, and the colon, liver and cecum were subsequently collected. The collected organs were washed with PBS solution, carefully blot-dried with filter paper and preserved at −80 °C until further analysis. Distal colon tissue near the anal portion was fixed with 4% paraformaldehyde for subsequent analysis. The entire blood sample was centrifuged at 4000× *g* for 15 min at 4 °C to collect serum, which was kept frozen at −80 °C until further analysis. All mice were anesthetized with isoflurane before dissection. The animal experiment was approved by Jiangxi University of Chinese Medicine Experimental Animal Science and Technology Center (JZLLSC20220492, 29 March 2022).

### 2.5. Hematoxylin and Eosin (H&E) Staining and Immunofluorescence Staining

After the colonic tissue was completely fixed with 4% paraformaldehyde, it was embedded in paraffin and sliced into sections with a thickness of 5 μm before being dewaxed. Then, the sections were stained using hematoxylin and eosin, and the resulting stain observations were photographed and analyzed with an inverted microscope. The detection of the tight junction protein (ZO-1) in colonic tissues was conducted through immunohistochemistry [26]. The fixed colon tissue was sectioned and dewaxed, and then the antigen repair and BSA closure were performed. Subsequently, the slices were then incubated with the primary antibody specific for the target (ZO-1), followed by the secondary antibody labelled with fluorescence. Finally, the sliced sections were analyzed under a fluorescence microscope and their fluorescence intensity was measured through ImageJ software 1.53v (National institutes of health, Bethesda, MD, USA). 

### 2.6. Inflammatory Cytokine Measurement in the Colon

The homogenized colon tissue was centrifuged, and the supernatant after centrifugation was used for detection. The levels of interleukin-6 (IL-6), tumor necrosis factor-alpha (TNF-α), interleukin-1 beta (IL-1β), and interleukin-10 (IL-10) in the colon tissue of mice were determined by a double-antibody sandwich method employing enzyme-linked immunosorbent assay (ELISA) kits (MEIMIAN, Yancheng, China) according to the given instructions.

### 2.7. Real-Time Quantitative Polymerase Chain Reaction (RT-qPCR)

Total RNA was extracted from colon tissues using the RNAeasy™ Animal RNA Isolation Kit with Spin Column (Beyotime, Shanghai, China), following the protocol of the kit. Briefly, the homogenized colon tissue was lysed and then centrifuged to take the supernatant, and finally total RNA was collected by the centrifugal column method. Then, we used a cDNA reverse transcription kit to reverse-transcribe the extracted RNA into cDNA. After that, TB Green™ Premix Ex Taq™ II (TliRNaseH Plus) (TaKaRa, Beijing, China) was added into the cDNA and quantified using the CFX Connect™ Fluorescent quantitative PCR detection system (BIO-RAD, Hercules, CA, USA). The related gene expression levels were objectively calculated using 2^−ΔΔCt^ method, while beta-actin was used as the internal reference gene. Table S2 provides the primer sequence list of ZO-1, Claudin-1, Occludin, MAPK p38, JNK, NF-κB p65 and IκB-α.

### 2.8. Determination of Content of Short-Chain Fatty Acids (SCFAs)

The extraction of short-chain fatty acids (SCFAs) was carried out according to the previously reported method with slight adaptations [27]. Fecal samples (50 mg) were mixed with deionized water (0.35 mL) and 10% sulfuric acid (17.5 µL) and sonicated in an ice bath for 10 min. After allowing mixture to stand for 20 min, the supernatant was collected by centrifugation at 10,000× *g* for 15 min at 4 °C. Then, the precipitate was mixed with 0.35 mL of deionized water and 17.5 µL of 10% sulfuric acid; the above operation was repeated once and the supernatant was combined. The supernatant was subsequently filtered through a 0.22 µm microporous membrane for the follow-up gas chromatography (GC) analysis. The gas chromatographic conditions were slightly adapted with reference to the method reported previously. The type of gas chromatographic column was an Agilent DB-WAX capillary column (30 m × 0.25 mm × 0.25 μm), and the temperature procedure was the same as the method of Guo et al. [6]. Finally, a corresponding standard curve was established to quantify the content of SCFAs in fecal specimens.

### 2.9. 16S rRNA Sequencing of Microbial Flora from the Cecal Feces

For 16S rRNA gene sequencing, the cecum content specimens were sent to Shanghai Majorbio Bio-pharm Technology Co., Ltd. (Shanghai, China). Subsequently, the sequencing results were analyzed via the Majorbio company’s cloud platform (www.MajorBio.com, accessed on 25 July 2023).

### 2.10. Statistical Analysis

All biochemical measurements and data obtained from reverse transcription polymerase chain reaction (RT-PCR) were analyzed using GraphPad 9.0.0 software. Significant differences among different groups in the 16S rRNA sequencing data were analyzed using a Kruskal–Wallis or Wilcoxon rank sum test. For multiple comparison tests, Duncan’s method was employed following a one-way ANOVA. SPSS software (version 19.0, SPSS, Chicago, IL, USA) was used for data analysis of the gut microbiota correlation. All data were presented as the mean ± standard error of the mean (SEM), with statistical significance being defined as *p* < 0.05.

## 3. Results

### 3.1. The Polyphenols Composition of WBP

The UPLC-ESI-QTOF-MS^2^ analysis of WBP tentatively identified 10 key compounds by combining literature reports and matching databases. These results are presented in Table 1, with phenolic acids identified as the primary compounds in WBP. Compound 3 had an *m*/*z* of 169.0142 for [M−H]^−^ in the negative ion mode, with a typical fragment ion of *m*/*z* 125.0234 [M−H−CO_2_]^−^, which was identified as gallic acid according to the literature report [28]. Compound 4 had an *m*/*z* of 153.0183 for [M−H]^−^ in the negative ion mode, with a typical fragment ion of *m*/*z* 109.0291 [M−H−CO_2_]^−^, which was presumed to be protocatechuic acid. Compound 5 exhibited an *m*/*z* of 179.0341 for [M−H]^−^ in negative ion mode, and its typical fragment ion was *m*/*z* 135.0443 [M−H−CO_2_]^−^, which was presumed to be caffeic acid based on the literature [29]. Compound 6 was identified as vanillic acid by its [M−H]^−^ *m*/*z* 167.0321 and fragment ions at 152.0121 [M−H−CH_3_]^−^ and 108.0231 [M−H−CH_3_−CO_2_]^−^, while compound 9 was identified as ferulic acid by its [M−H]^−^ *m*/*z* 193.0494 and fragment ions at 178.0261 [M−H−CH_3_]^−^ and 149.0603 [M−H−CO_2_]^−^ [30]. Their secondary mass spectra are shown in Figure S1.

**Figure 2:**
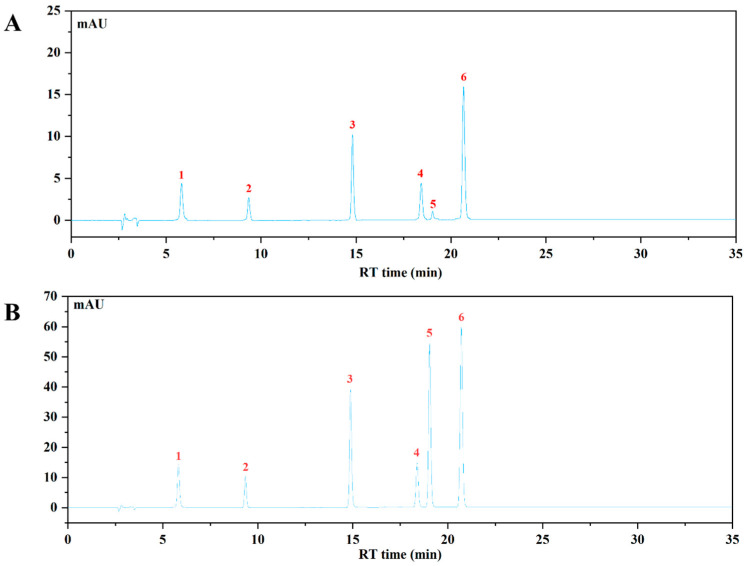
The HPLC chromatogram of WBP and mixed standards using VWD detection at 320 nm. (**A**) The HPLC profile of WBP; (**B**) the HPLC profile of mixed standards: (1) gallic acid, (2) protocatechuic acid, (3) caffeic acid, (4) vanillic acid, (5) *p*-coumaric acid, (6) ferulic acid.

The HPLC-VWD analysis (Figure 2) suggested that the major six phenolic compounds were gallic acid (138.15 ± 0.24 μg/g), protocatechuic acid (84.09 ± 0.95 μg/g), caffeic acid (115.02 ± 0.47 μg/g), vanillic acid (176.59 ± 1.59 μg/g), *p*-coumaric acid (6.36 ± 0.15 μg/g), and ferulic acid (159.86 ± 1.11 μg/g). All of these compounds were identified and quantified by comparing with their respective reference standards (Table 2).

### 3.2. WBP Alleviated the Symptoms of UC Induced by DSS in Mice

**Figure 3:**
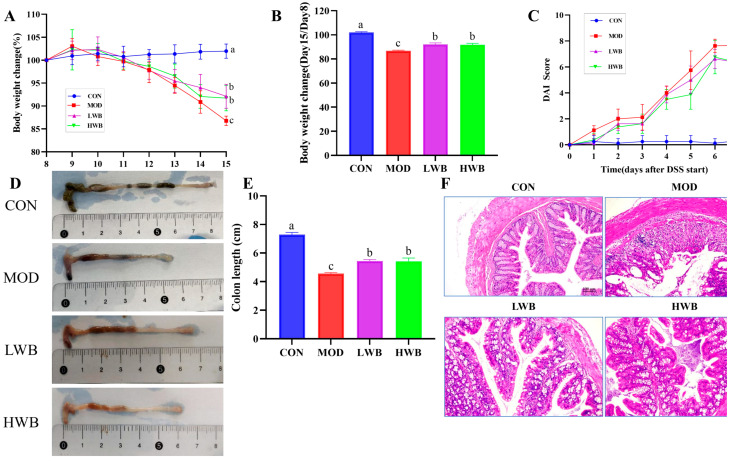
Anti-inflammatory activity of WBP in vivo. (**A**) daily weight change; (**B**) the change of BW in different groups (Day 15/Day 8); (**C**) DAI score results; (**D**) photos of mouse colon in different groups; (**E**) the results of colon length; (**F**) the H&E-stained histopathological sections of colonic tissues. Data are presented as mean ± SEM (*n* = 8). Significant differences are indicated by different letters (*p* < 0.05).

After administration of DSS, the general condition and the DAI of the mice were assessed. As presented in Figure 3A, the mice in the CON group showed a gradual increase in body weight (BW), and the increase was not significant. Conversely, the other three groups showed a significant decrease (*p* < 0.01) in BW after DSS treatment according to Figure 3B. Specifically, the weight of the MOD group, LWB group, and HWB group decreased by 12.7%, 8.7%, and 9.0%, respectively. Notably, the rate of weight loss was significantly reduced in both the LWB group (*p* < 0.01) and the HWB group (*p* < 0.01) compared with the MOD group. Meanwhile, the DAI scores were significantly decreased in the HWB group (6.33 ± 0.52, *p* < 0.05) and the LWB group (6.33 ± 1.03, *p* < 0.05) compared to the MOD group (7.67 ± 0.21), indicating a decrease in disease severity, as shown in Figure 3C. These results indicate that dietary intake of WBP can alleviate the symptoms of UC caused by DSS.

### 3.3. WBP Reduced the Colon Tissue Damage in Mice with Colitis

Histological analysis was performed to examine the histopathological condition of the colon. As shown in Figure 3D,E, the mean length of the colon in the CON group was 7.29 ± 0.37 cm. The colonic length in the two dosage groups of WBP was 5.44 ± 0.26 cm (LWB) and 5.42 ± 0.59 cm (HWB), respectively, which was significantly greater than that in the MOD group, which measured 4.56 ± 0.19 cm (*p* < 0.01). H&E staining (Figure 3F) of the distal colon showed that the colon sections of mice in the CON group showed an intact epithelial cell surface with a clear tissue structure, and the mucosal layer and crypts were visible. However, the intake of DSS led to the disruption of the normal colon structure, resulting in an increased infiltration of inflammatory cells. This in turn caused extensive surface epithelial cell erosion, mucosal damage and crypt distortion. Notably, in comparison to the MOD group, the LWB and HWB groups exhibited a noticeable improvement in the structure of the epithelial recess, indicating partial repair. Furthermore, the severity of inflammation was reduced in these groups. To summarize, the administration of WBP was found to mitigate DSS-caused colon tissue damage in mice by promoting an increase in colon length and preserving colon tissue structure.

### 3.4. WBP Regulated the Levels of Inflammatory Cytokines in Colonic Tissues

**Figure 4:**
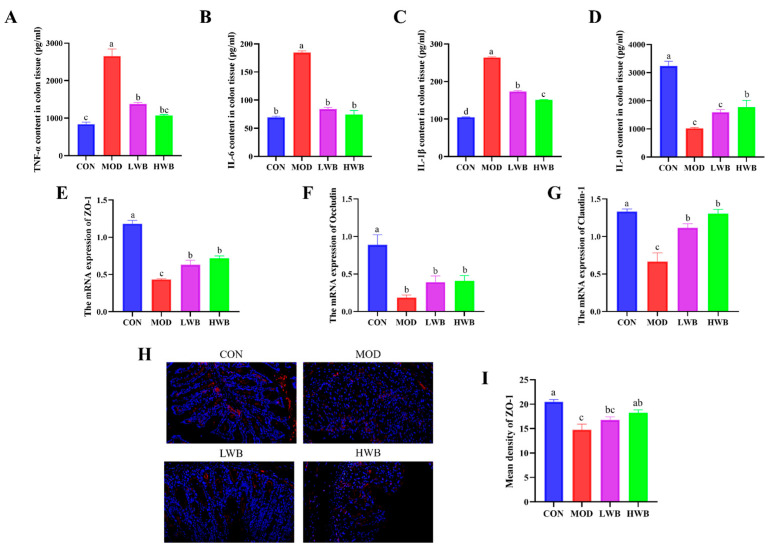
Levels of colonic inflammatory indicators and the expression of TJ proteins. (**A**–**D**) The inflammatory cytokine expression level of TNF-α, IL-6, IL-1β and IL-10 in the colons; (**E**–**G**) the mRNA expression level of TJ protein ZO-1, Occludin, and Claudin-1; (**H**) fluorescence image of ZO-1 at 200× magnification (scale bars = 100 μm), with the target protein expressed as red fluorescence; (**I**) fluorescence quantitative results of ZO-1 protein. Significant differences between groups are indicated by different letters in each column (*p* < 0.05).

The impact of WBP on the severity of colonic inflammation was investigated by detecting the expression of inflammatory factors (TNF-α, IL-1β, IL-6, IL-10) in colonic tissue using ELISA kits. The expression of pro-inflammatory cytokines (TNF-α, IL-6, and IL-1β) in colon tissues were found to be upregulated significantly in the MOD group compared to the CON group (*p* < 0.0001), as demonstrated in Figure 4A–D. After the treatment with WBP, the expression of these three pro-inflammatory cytokines was remarkably downregulated in both dose groups (*p* < 0.001). In contrast, the expression of the anti-inflammatory cytokine IL-10 in colon tissues was significantly downregulated in the MOD group (*p* < 0.001), and WBP was able to inhibit this change. However, only the high-dose group exhibited a significant effect on the expression of IL-10 (*p* < 0.05). The HWB group demonstrated a superior anti-inflammatory activity compared to the LWB group in terms of regulating colonic inflammatory factors. In summary, WBP has the ability to alleviate colonic inflammation in mice by regulating expression of inflammatory factors.

### 3.5. WBP Improved the Intestinal Barrier Function in Mice

To estimate the protective effect of WBP, the gene expressions of tight junction (TJ) proteins (ZO-1, Occludin, Claudin-1) were examined. Figure 4E–G demonstrates a significant decrease in the mRNA expression of these three TJ proteins in the MOD group (*p* < 0.01), suggesting severe damage to intestinal barrier function after DSS treatment. Intragastric administration of WBP effectively reversed this phenomenon in mice. The mRNA expression of ZO-1 and Claudin-1 was significantly increased in both the LWB (*p* < 0.05) and HWB (*p* < 0.01) groups, compared with the MOD group. In the immunofluorescence result of ZO-1 protein (Figure 4H,I), the high dose of WBP (HWB) significantly enhanced the expression of ZO-1 protein (*p* < 0.01). In conclusion, WBP improves the intestinal barrier function in mice by increasing TJ protein expression, especially ZO-1. Moreover, the HWB group demonstrates a more pronounced effect in enhancing the expression level of TJ proteins compared to the LWB group.

### 3.6. WBP Regulated the Conduction of NF-κB and MAPK Signaling Pathways

**Figure 5:**
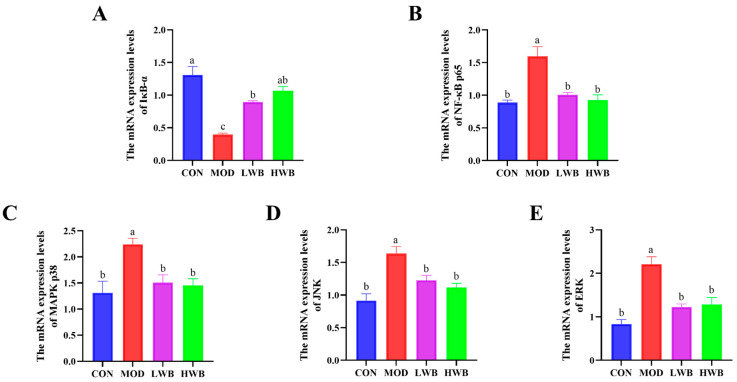
The mRNA expression level of inflammatory pathway-related proteins. (**A**,**B**) The mRNA expression levels of NF-κB-related pathway proteins IκB-α and p65; (**C**–**E**) the mRNA expression levels of MAPK-related pathway proteins JNK, ERK, and p38. Data are presented as mean ± SEM (*n* = 6). Significant differences between groups are indicated by different letters in each column (*p* < 0.05).

Figure 4B displays the expression of the mRNA level of NF-κB p65 in the control group (CON) and the two WBP dose groups (LWB: 1.01 ± 0.03 and HWB: 0.93 ± 0.08). Both WBP dose groups exhibited a remarkably (*p* < 0.01) lower mRNA expression than the MOD group (1.59 ± 0.15). Conversely, there was a significant decrease (*p* < 0.001) in the mRNA expression of IκB-α—a vital protein that regulates the NF-κB signaling pathway—in the MOD group in comparison to the CON group. Nevertheless, after treatment with WBP, the mRNA expression of IκB-α was significantly upregulated in both the LWB (*p* < 0.01) and HWB (*p* < 0.001) groups (Figure 5A,B). As for the MAPK signaling pathway (Figure 5C–E), the mRNA expression levels of p38 (*p* < 0.05), JNK, and ERK (*p* < 0.001) were remarkably downregulated in the MOD group after treatment with DSS compared to the CON group. However, in the LWB and HWB groups, WBP administration attenuated the downregulation of gene expression of these three MAPK pathway-related proteins in the MOD group. Notably, in the LWB group, the mRNA expression of ERK (*p* < 0.01), JNK, and p38 (*p* < 0.05) was substantially higher compared to the MOD group. Additionally, the HWB group exhibited a more significant beneficial effect on the mRNA expression of JNK (*p* < 0.01), and its treatment effects on the other two proteins were consistent with the LWB group. These findings indicate that WBP exerts its anti-inflammatory effect via modulating the expression of related genes in pathways associated with inflammation. Furthermore, a higher dose of WBP may yield more favorable outcomes.

### 3.7. WBP Regulated the Production of SCFAs in Cecal Contents

**Figure 6:**
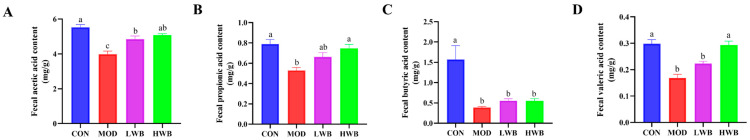
Analysis for SCFAs in feces. (**A**) The content of acetic acid in feces; (**B**) the content of propionic acid in feces; (**C**) the content of butyric acid in feces; (**D**) the content of valeric acid in feces. Data are presented as mean ± SEM (*n* = 6). Significant differences are indicated by different letters (*p* < 0.05).

Figure 6A–D shows that the MOD group had significantly lower levels of acetic acid (3.98 ± 0.18 mg/g), propionic acid (0.53 ± 0.03 mg/g), butyric acid (0.39 ± 0.03 mg/g), and valeric acid (0.17 ± 0.01 mg/g) compared to the CON group (*p* < 0.01). Nevertheless, the supplementation with high doses of WBP (HWB) significantly reduced the levels of valeric acid (*p* < 0.001), acetic acid, and propionic acid (*p* < 0.01). In the LWB group, the content of acetic acid was significantly improved (*p* < 0.05). In summary, the supplementation of WBP can help maintain proper levels of intestinal SCFAs in UC mice.

### 3.8. WBP Attenuates Disturbances in the Gut Microbiota

**Figure 7:**
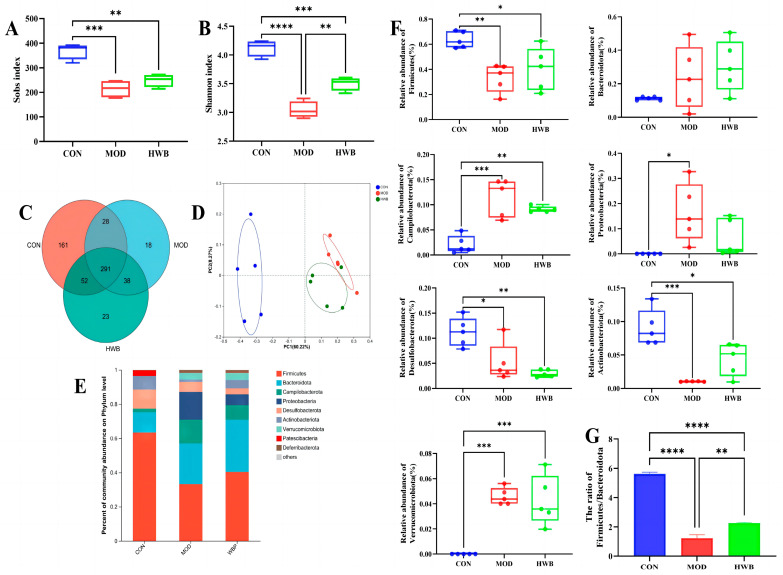
The impact of WBP on the modulation of intestinal flora composition in colitis mice induced by DSS. (**A**,**B**) The Sobs and Shannon indices; (**C**) Venn diagram of common and unique OTUs among different groups; (**D**) PCoA analysis of intestinal microbiota in different groups; (**E**) top ten microbial genera in terms of abundance at the phylum level; (**F**) the abundance of differential microbiota at the phylum level in three groups; (**G**) the ratio of *Firmicutes/Bacteroidota* in different groups. Data are presented as mean ± SEM (*n* = 5). * *p* < 0.05, ** *p* < 0.01, *** *p* < 0.001 and **** *p* < 0.0001.

Intestinal inflammation onset and progression are significantly influenced by dysbiosis of the intestinal microbiota [31]. As depicted in Figure 7A,B, compared with the CON group, the Sobs index (*p* < 0.001) and the Shannon index (*p* < 0.0001), which are connected with the alpha diversity of intestinal microbiota, were significantly lower following DSS treatment. WBP supplementation could significantly increase the Shannon index (*p* < 0.01). In Figure 7C, the Venn diagram showed that 611 Operational Taxonomic Units (OTUs) were identified from three groups (*n* = 5), of which 291 OTUs were mutual. The unique OTU counts were as follows: 161 in the CON group, 18 in the MOD group, and 23 in the HWB group. Principal co-ordinates analysis (PCoA) revealed that both the MOD and HWB groups were obviously separated from the CON group (Figure 7D).

At the phylum level, the intake of DSS resulted in a significant increase in the abundance of *Proteobacteria* (*p* < 0.05), *Campilobacterota* and *Verrucomicrobiota* (*p* < 0.001), and a reduction in the abundance of *Desulfobacterota* (*p* < 0.05), Firmicutes (*p* < 0.01), and *Actinobacteriota* (*p* < 0.001) (Figure 7E,F). The supplementation of WBP effectively suppresses these variations induced by DSS except for the *Desulfobacterota*. The ratio of *Firmicutes/Bacteroidota* exhibited a considerable reduction (*p* < 0.0001) in the MOD group when compared with the CON group (Figure 7G). In contrast, after treatment with WBP, such a ratio was remarkably increased (*p* < 0.01).

**Figure 8:**
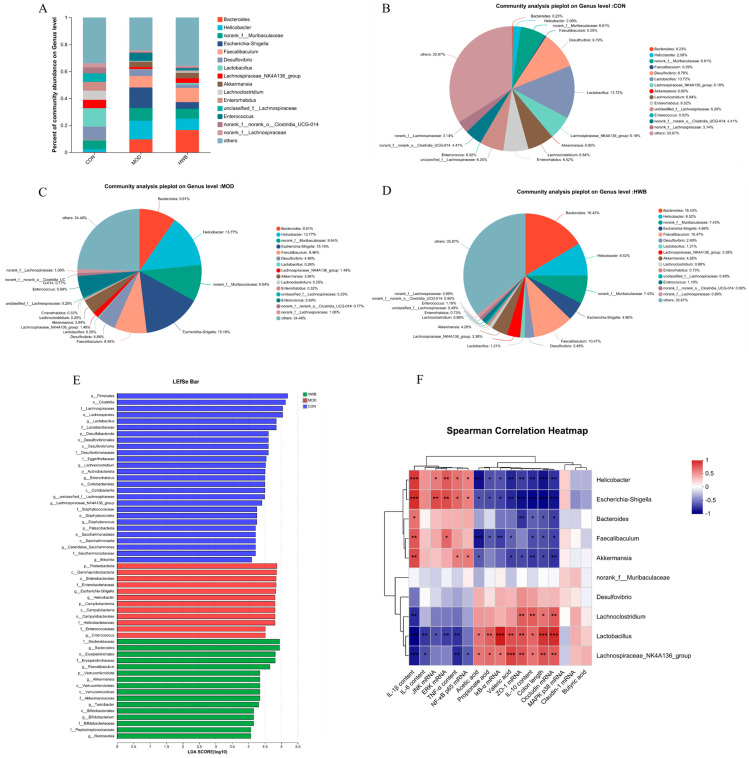
The impact of WBP supplementation on the abundance of gut microbiota at the genus level. (**A**) Genus level distribution histogram of the gut microbiota, (**B**–**D**) distribution pie chart of genus levels in CON, MOD, and HWB groups; (**E**) score for abundances of different taxa using linear discriminant analysis (LDA); (**F**) Spearman’s correlation analysis between gut microbiota at the genus level and various indices. The correlation between variables is indicated by the color red for positive and blue for negative, * *p* < 0.05, ** *p* < 0.01 and *** *p* < 0.001.

At the genus level, induction of DSS resulted in a rise in the relative abundance of *Bacteroides*, *Helicobacter*, *norank_f_Muribaculaceae*, *Escherichia-Shigella*, *Faecalibaculum*, *Akkermansia,* and *Enterococcus* compared to the CON group, increasing from 0.23% to 9.61%, 2.08% to 13.77%, 6.61% to 9.54%, 0% to 15.19%, 0.29% to 8.46%, 0 to 4.58%, and 0.02% to 5.69%, respectively (Figure 8A–D). Supplementation with WBP reversed the enhancement in abundance of these bacterial genera except for the *Bacteroides*. Meanwhile, the abundance of *Desulfovibrio*, *Lactobacillus*, *Lachnospiraceae_NK4A136_group*, *Lachnoclostridium*, *Enterorhabdus*, *unclassified_f_Lachnospirac*, *norank_f_norank_o_Clostridia_UCG-014,* and *norank_f_Lachnospiraceae* was decreased in the MOD group. Moreover, the supplementation of WBP could reverse these changes.

LEfSe (linear discriminant analysis effect size) analysis revealed those microbial communities that significantly discriminated between different groups (*p* < 0.05, LDA score (log10) > 4, Figure 8E). Among the examined groups, a total of 65 distinct taxa (from phylum to genus levels) were identified; 12 and 16 bacterial genera were enriched in the MOD and HWB groups, respectively. *p_Proteobacteria*, *c_Gamma-proteobacteria,* and *o_Emterobacterales* were the three most enriched bacteria in the MOD group, while *o_Erysipelotrichales* was enriched in the HWB group.

To investigate the connection between gut microbiota and colitis phenotype in mice, Spearman’s correlation analysis was employed. Figure 8F illustrates that both the *Escherichia-Shigella* and the *Helicobacter* exhibited significant negative correlations (*p* < 0.05) with colon length, IκB-α mRNA, SCFAs (acetic acid, propionate acid, and valeric acid), TJ proteins (ZO-1, Occludin), and anti-inflammatory cytokine content (IL-10). Additionally, a significant positive correlation was observed (*p* < 0.05) between their expression of mRNA JNK, ERK, NF-κB, and p65, and their content of pro-inflammatory factors (TNF-α, IL-1β). Interestingly, the *Lactobacillus* and *Lachnospiraceae_NK4A136_group* showed an opposite result compared to the *Escherichia-Shigella* and the *Helicobacter*.

## 4. Discussion

UC is a chronic condition that causes inflammation in the bowels and disrupts the balance of microbial in the gut. UC occurs in individuals of all age groups and poses a global public health challenge [32]. At present, there is an urgent need for alternative treatment methods due to side effects and high drug resistance of the medicines used to treat colitis [33]. Therefore, more and more studies have focused on natural plant compounds with multiple biological activities and minimal side effects. Polyphenols have also gained significant attention as natural plant compounds and are considered potential agents for treating UC. Many researchers have indicated that phenolic acids, as one of the bioactive phytochemicals, have potent anti-inflammatory and antioxidant activities [34,35,36]. The results of the WBP characterization revealed that the polyphenols present in wheat bran consisted primarily of phenolic acids, including ferulic acid, caffeic acid, gallic acid, vanillic acid, protocatechuic acid, and *p*-coumaric acid. Maryam et al. found that ferulic acid could ameliorate UC in rats by the inhibition of the LPS-TLR4-NF-κB and the NF-κB-INOS-NO signaling pathways [37]. Meghna et al. reported that vanillic acid exerted its anti-inflammatory activity via regulating the IKK-NF-κB pathway [38]. Zhu Lei et al. also discovered that gallic acid exhibited protective properties against TNBS-triggered colitis through inflammation inhibition and apoptosis stimulation via the NF-κB pathway [39]. Danuta et al. indicated that caffeic acid could regulate processes related to intestinal inflammation [40]. Moreover, several research have found that protocatechuic acid and *p*-coumaric acid possess antioxidant and anti-inflammatory properties [41,42], although they were less abundant in WBP. Building on previous research reports and the identification of phenolic substances in WBP, we suggest that WBP holds the potential as a palliative intervention for UC.

The results of animal experiments showed that dietary supplementation of WBP effectively alleviated colitis symptoms induced by DSS. Additionally, staining results of mouse colonic tissue sections demonstrated that WBP can alleviate colonic inflammation by restoring crypt shape, reducing of inflammatory cell infiltration, and relieving damaged colonic tissue. Damage to the intestinal barrier is strongly linked to UC, evidenced by the reduction or even disappearance of TJ proteins, disrupted distribution, and heightened intestinal mucosal epithelial cell permeability. This ultimately leads to an increased penetration of harmful bacteria and toxins in the colon, resulting in symptoms such as diarrhea, shortened colon length, and colonic bleeding [43,44,45]. This study found that both LWB and HWB could alleviate the reduced mRNA expression levels of tight junction proteins (ZO-1, Claudin-1, and Occludin) present in the colonic tissue of mice induced with colitis. Furthermore, immunofluorescence analysis revealed that a high dose of WBP (HWB) significantly enhanced ZO-1 protein expression. Overall, WBP exerts a protective impact on the colonic mucosa by preserving TJ protein expression in the colon, resulting in symptom relief in colitis mice induced by DSS.

Previous research has indicated that overexpression of TNF-α and IL-1β may impair the integrity of the intestinal tight junction barrier [46,47]. The release of various inflammatory factors, including anti-inflammatory and pro-inflammatory factors, is regarded as a key pathophysiological indicator of UC [48]. Clinical studies have demonstrated that the severity of UC is determined by the imbalance of anti-inflammatory and pro-inflammatory factors, while the overexpression of pro-inflammatory cytokines could lead to mucosal inflammation in the intestines [49]. TNF-α plays a crucial role in promoting intestinal epithelial mucosal injury in colitis as a pro-inflammatory factor that initiates an immune response to harmful stimuli. In addition, TNF-α stimulates the synthesis of the pro-inflammatory cytokines IL-6 and IL-1β, intensifying and aggravating the inflammatory reaction [27]. IL-10, a significant anti-inflammatory cytokine, decreases mouse colitis through inhibiting the production of pro-inflammatory cytokine and suppressing the inflammation in the intestines [50]. In this study, both the HWB and LWB treatments demonstrated a significant reduction in pro-inflammatory factors (TNF-α, IL-6, and IL-1β) in the colon tissues of colitis mice. Furthermore, these treatments increased the secretion of anti-inflammatory factors (IL-10). Moreover, HWB had a more protective effect against DSS-induced UC when compared to LWB.

To further investigate the mechanism of action by which WBP relieves colonic inflammation, we investigated the involvement of the NF-κB and MAPK signaling pathways to inflammation regulation. NF-κB functions as a transcription factor that forms a heterodimer with the p65/p50 subunit and inhibitory protein IκB within the cytoplasm [51]. Upon activation of the signaling pathway, the degradation of IκB proteins enables the translocation of NF-κB dimers into the nucleus, thereby regulating the expression of target genes [52]. Additionally, several studies have indicated that an excess of TNF-α can stimulate the NF-κB signaling pathway [48]. In our investigation, there was a notable enhancement in mRNA expression of NF-κB p65, accompanied by a notable reduction in the expression of IκB-α in colitis mice. These findings propose an activated NF-κB signal pathway in comparison to the CON group. However, after treatment with both LWB and HWB, the mRNA expression levels of NF-κB, p65, and IκB-α were significantly reversed, indicating that WBP possesses the ability to hinder the stimulation of the NF-κB pathway. MAPKs, a cluster of protein serine/threonine kinases such as ERK, JNK, and p38, have a crucial function in the synthesis of inflammatory cytokines in mammals [53]. Various researchers have illustrated that the MAPK signaling pathway induces the secretion and gene expression of some pro-inflammatory cytokines, such as TNF-α, IL-6, and IL-1β, thereby intensifying the inflammatory response [27,54]. In this study, both the LWB and HWB treatments were found to inhibit the activation of the MAPK pathway by reducing the mRNA expression of JNK, ERK, and p38. In summary, the administration of WBP displayed inhibitory properties on activating the NF-κB and MAPK signaling pathways, which subsequently attenuated intestinal inflammation by mitigating the secretion and expression of pro-inflammatory cytokines.

Numerous researchers have shown that plant polyphenols could uphold the proper functioning of the gut barrier by interacting with the gut microbiota [55]. SCFAs, as one of the primary beneficial metabolites of intestinal flora, have the ability to exhibit anti-inflammatory activity and protect gut barrier integrity through interaction with G-protein-coupled receptors such as GPR109A, GPR43, and GPR41 [56]. Furthermore, the increase in SCFA production acidifies the intestinal environment, enhances nutrient absorption, and inhibits pathogen growth [57]. In this study, a noteworthy reduction in SCFAs levels, such as acetic acid, propionic acid, butyric acid, and valeric acid, was noted after administering DSS. The observations were deemed significant. However, both the LWB and HWB groups were able to increase SCFAs content through treatment. Fernando et al. found that *Lactobacillus* has been linked to the biosynthesis of acetic acid and plays a big part in maintaining intestinal barrier function [58]. *Lachnospiraceae_NK4A136_group* is one of the butyric acid producers in the gut essential in maintaining immune homeostasis [1,59,60]. After the WBP treatment, the abundance of these two genera was augmented in mice experiencing DSS-induced colitis. Moreover, previous research has found a correlation between UC and an imbalance of intestinal flora [3,7]. The results of 16S rRNA sequencing indicated that the Sobs and Shannon indices, which describe the α diversity of microflora, were larger in the HWB group compared with the MOD group. This indicates that WBP can regulate intestinal health by maintaining the richness and diversity of the gut microbiome. In addition, the PCoA results suggested that the gut microflora composition of the HWB group shared greater similarity with that of the CON group. At the phylum level, *Firmicutes* and *Bacteroidetes* are the two most dominant phyla, accounting for more than 60% of the entire microbiome. The *Firmicutes/Bacteroidetes* ratio was significantly reduced in the MOD group and had a marked increase post the WBP intervention. Stojanov et al. found a decreased *Firmicutes/Bacteroidetes* ratio in patients with IBD [61]. Flaviana et al. found that *Escherichia-Shigella* intensifies intestinal inflammation by secreting IL-6 and TNF-α [62]. Peng et al. identified *Helicobacter* as key bacteria in colitis [63]. *Akkermansia* is a mucin-degrading bacterium of the intestinal mucosa. Zhou et al. reported that the excessive proliferation of *Akkermansia* in the gut leads to the direct exposure of the intestinal surface to harmful pathogens (virus, pathogenic bacteria, etc.) after consuming a large amount of mucosal protein, which may induce UC [4]. Meanwhile, Zou et al. found that *Lachnospiraceae_NK4A136_group* functions as a probiotic, promoting immune homeostasis by regulating the Th17/Treg balance. *Lactobacillus* is a well-recognized beneficial bacterium that is utilized for immune system regulation and the treatment of gastrointestinal disorders. Numerous studies have demonstrated that supplementing with different strains of *Lactobacillus* can mitigate DSS-induced colitis [64,65,66,67]. In our study, there was a noteworthy enhancement in the abundance of *Helicobacter*, *Escherichia-Shigella*, and *Akkermansia* subsequent to DSS administration in colitis-afflicted mice, while the abundances of *Lachnospiraceae_NK4A136_group* and *Lactobacillus* were reduced. However, in the HWB group, the results demonstrated that WBP could reverse these changes. In summary, the above results demonstrate that WBP has the ability to regulate the intestinal microbiota through augmenting the prevalence of advantageous bacteria whilst diminishing the frequency of harmful bacteria. This modulation leads to higher levels of SCFAs, ultimately regulating intestinal inflammation.

## 5. Conclusions

In conclusion, this study suggests that the WBP contains abundant phenolic acids including ferulic acid, caffeic acid, vanillic acid, and gallic acid. The administration of WBP reduces inflammation in the colon by inhibiting the overexpression of NF-κB and MAPK-related inflammatory signaling pathways. Furthermore, WBP maintains the normal function and permeability of the intestinal barrier by sustaining the integrity of the intestinal epithelial mucosa. Our results suggest that WBP has excellent potential in combating ulcerative colitis.

## Figures and Tables

**Table 1:** Polyphenol composition identified in WBP (wheat bran).

Number	t_R_(min)	*m*/*z*	Formula	Type	Major Fragment	Identification
1	0.749	191.0185	C_6_H_8_O_7_	[M-H]^−^	85.0294; 111.0088; 173.0082	Citric acid
2	2.425	203.0819	C_11_H_12_N_2_O_2_	[M-H]^−^	116.0503; 142.0652	L-Tryptophan
3	3.141	169.0142	C_7_H_6_O_5_	[M-H]^−^	125.0234	Gallic acid
4	4.276	153.0183	C_7_H_6_O_4_	[M-H]^−^	109.0291	Protocatechuic acid
5	4.667	179.0341	C_9_H_8_O_4_	[M-H]^−^	135.0443;	Caffeic acid
6	5.262	167.0321	C_8_H_8_O_4_	[M-H]^−^	108.0231; 152.0121	Vanillic acid
7	5.824	163.0388	C_9_H_8_O_3_	[M-H]^−^	119.0491;	*p*-Coumaric acid
8	6.352	563.1381	C_26_H_28_O_14_	[M-H]^−^	353.0677; 383.0768; 473;1098	Isoschaftoside
9	6.706	193.0498	C_10_H_10_O_4_	[M-H]^−^	149.0603; 178.0261	Ferulic acid
10	9.651	579.1714	C_27_H_32_O_14_	[M-H]^−^	271.0607	Narirutin

**Table 2:** The contents of individual phenolic acid compounds in WBP extracts.

Num.	Compound	Molecular Formula	Standard Curve	Correlation Coefficient	Content (μg/g)
1	Gallic acid	C_7_H_6_O_5_	y = 0.0031x − 0.0072	0.9993	138.15 ± 0.24
2	Protocatechuic acid	C_7_H_6_O_4_	y = 0.0015x − 0.0207	0.9996	84.09 ± 0.95
3	Caffeic acid	C_9_H_8_O_4_	y = 0.0004x − 0.0014	0.9986	115.02 ± 0.47
4	Vanillic acid	C_8_H_8_O_4_	y = 0.0055x − 0.0051	0.9996	176.59 ± 1.59
5	*p*-Coumaric acid	C_9_H_8_O_3_	y = 0.0002x − 0.0025	0.9990	6.36 ± 0.15
6	Ferulic acid	C_10_H_10_O_4_	y = 0.0002x − 0.0024	0.9993	159.86 ± 1.11

Data are expressed as mean ± SEM of three independent samples. Each measurement procedure was performed in triplicate.

## Data Availability

Data is contained within the article or supplementary material.

## Supplementary Materials

The following supporting information can be downloaded at: https://www.mdpi.com/article/10.3390/foods13020225/s1, Figure S1: MS/MS spectra and fragmentation patterns of selected phenolic components; Table S1: Disease activity index (DAI) scoring criteria; Table S2: Primer sequence for quantitative PCR.

## References

[1] Zou M., Wang Y., Liu Y., Xiong S., Zhang L., Wang J. (2023). Huangshan Floral Mushroom Polysaccharide Ameliorates Dextran Sulfate Sodium-Induced Colitis in Mice by Modulating Th17/Treg Balance in a Gut Microbiota-Dependent Manner. Mol. Nutr. Food Res..

[2] Yadav V., Varum F., Bravo R., Furrer E., Bojic D., Basit A.W. (2016). Inflammatory bowel disease: Exploring gut pathohysiology for novel therapeutic targets. Transl. Res..

[3] Guo X.Y., Liu X.J., Hao J.Y. (2020). Gut microbiota in ulcerative colitis: Insights on pathogenesis and treatment. J. Dig. Dis..

[4] Zhou Z., He W., Tian H., Zhan P., Liu J. (2023). Thyme (*Thymus vulgaris* L.) polyphenols ameliorate DSS-induced ulcera-tive colitis of mice by mitigating intestinal barrier damage, regulating gut microbiota, and suppressing TLR4/NF-κB-NLRP3 inflammasome pathways. Food Funct..

[5] Sartor R.B., Wu G.D. (2017). Roles for Intestinal Bacteria, Viruses, and Fungi in Pathogenesis of Inflammatory Bowel Diseases and Therapeutic Approaches. Gastroenterology.

[6] Guo F., Tsao R., Li C., Wang X., Zhang H., Jiang L., Sun Y., Xiong H. (2021). Green Pea (*Pisum sativum* L.) Hull Polyphenol Extracts Ameliorate DSS-Induced Colitis through Keap1/Nrf2 Pathway and Gut Microbiota Modulation. Foods.

[7] Kobayashi T., Siegmund B., Le Berre C., Wei S.C., Ferrante M., Shen B., Bernstein C.N., Danese S., Peyrin-Biroulet L., Hibi T. (2020). Ulcerative colitis. Nat. Rev. Dis. Primers.

[8] Patra A.K., Amasheh S., Aschenbach J.R. (2019). Modulation of gastrointestinal barrier and nutrient transport function in farm animals by natural plant bioactive compounds—A comprehensive review. Crit. Rev. Food Sci. Nutr..

[9] Xia X., Lin H., Luo F., Wu X., Zhu L., Chen S., Luo H., Ye F., Peng X., Zhang Y. (2022). Oryzanol Ameliorates DSS-Stimulated Gut Barrier Damage via Targeting the Gut Microbiota Accompanied by the TLR4/NF-κB/NLRP3 Cascade Response In Vivo. J. Agric. Food Chem..

[10] Xiao J. (2022). Recent advances on the stability of dietary polyphenols. Efood.

[11] Carmona-Hernandez J.C., Taborda-Ocampo G., Valdez J.C., Bolling B.W., González-Correa C.H. (2019). Polyphenol Ex-tracts from Three Colombian Passifloras (Passion Fruits) Prevent Inflammation-Induced Barrier Dysfunction of Ca-co-2 Cells. Molecules.

[12] Shanmugam S., Thangaraj P., Dos Santos Lima B., Trindade G.G.G., Narain N., Mara De Oliveira E Silva A., San-tin J.R., Broering M.F., Serafini M.R., Quintans-Júnior L.J. (2020). Protective effects of flavonoid composition rich P. subpeltata Ortega. on indomethacin induced experimental ulcerative colitis in rat models of inflammatory bowel diseases. J. Ethnopharmacol..

[13] Cardona F., Andrés-Lacueva C., Tulipani S., Tinahones F.J., Queipo-Ortuño M.I. (2013). Benefits of polyphenols on gut microbiota and implications in human health. J. Nutr. Biochem..

[14] Shigeshiro M., Tanabe S., Suzuki T. (2013). Dietary polyphenols modulate intestinal barrier defects and inflammation in a murine model of colitis. J. Funct. Food..

[15] Shi L., Lin Q., Yang T., Nie Y., Li X., Liu B., Shen J., Liang Y., Tang Y., Luo F. (2016). Oral administration of Lentinus edodes β-glucans ameliorates DSS-induced ulcerative colitis in mice via MAPK-Elk-1 and MAPK-PPARγ pathways. Food Funct..

[16] Mahmoud T.N., El-Maadawy W.H., Kandil Z.A., Khalil H., El-fiky N.M., El Alfy T.S.M.A. (2021). *Canna x generalis* L.H. Bailey rhizome extract ameliorates dextran sulfate sodium-induced colitis via modulating intestinal mucosal dysunction, oxidative stress, inflammation, and TLR4/ NF-ҡB and NLRP3 inflammasome pathways. J. Ethnopharmacol..

[17] Zhang W., Li Y., Xu J., Shi L., Chen L., Lu Y., Wu Q., Luo J., Chen Y. (2022). Armillariella tabescens methanol extract ameliorates ulcerative colitis via inhibiting TLR4/NF-κB and NLRP3 activation and mediating intestinal barrier integrity. J. Funct. Food..

[18] Kaulmann A., Bohn T., Rupasinghe V. (2016). Bioactivity of Polyphenols: Preventive and Adjuvant Strategies toward Re-ducing Inflammatory Bowel Diseases—Promises, Perspectives, and Pitfalls. Oxidative Med. Cell. Longev..

[19] Barroso E., Muñoz-González I., Jiménez E., Bartolomé B., Moreno-Arribas M.V., Peláez C., Del Carmen Mar-tínez-Cuesta M., Requena T. (2017). Phylogenetic profile of gut microbiota in healthy adults after moderate intake of red wine. Mol. Nutr. Food Res..

[20] Ritchie L.E., Sturino J.M., Carroll R.J., Rooney L.W., Azcarate-Peril M.A., Turner N.D. (2015). Polyphenol-rich sorghum brans alter colon microbiota and impact species diversity and species richness after multiple bouts of dextran sodium sulfate-induced colitis. Fems Microbiol. FEMS Microbiol. Ecol..

[21] Zhao H., Cheng N., Zhou W., Chen S., Wang Q., Gao H., Xue X., Wu L., Cao W. (2019). Honey Polyphenols Ameliorate DSS-Induced Ulcerative Colitis via Modulating Gut Microbiota in Rats. Mol. Nutr. Food Res..

[22] Wang G., Yu Y., Wang Y., Wang J., Guan R., Sun Y., Shi F., Gao J., Fu X. (2019). Role of SCFAs in gut microbiome and glycolysis for colorectal cancer therapy. J. Cell. Physiol..

[23] Cheng W., Sun Y., Fan M., Li Y., Wang L., Qian H. (2022). Wheat bran, as the resource of dietary fiber: A review. Crit. Rev. Food Sci. Nutr..

[24] Stevenson L., Phillips F., O’Sullivan K., Walton J. (2012). Wheat bran: Its composition and benefits to health, a European perspective. Int. J. Food Sci. Nutr..

[25] Suchowilska E., Bieńkowska T., Stuper-Szablewska K., Wiwart M. (2020). Concentrations of Phenolic Acids, Flavonoids and Carotenoids and the Antioxidant Activity of the Grain, Flour and Bran of *Triticum polonicum* as Compared with Three Cultivated Wheat Species. Agriculture.

[26] Qin N., Liu H., Cao Y., Wang Z., Ren X., Xia X. (2023). Polysaccharides from the seeds of *Gleditsia sinensis* Lam. attenuate DSS-induced colitis in mice via improving gut barrier homeostasis and alleviating gut microbiota dysbiosis. Food Funct..

[27] Li L., Qiu N., Meng Y., Wang C., Mine Y., Keast R., Guyonnet V. (2023). Preserved egg white alleviates DSS-induced colitis in mice through the reduction of oxidative stress, modulation of inflammatory cytokines, NF-κB, MAPK and gut microbiota composition. Food Sci. Hum. Wellness.

[28] Sun Y., Deng Z., Liu R., Zhang H., Zhu H., Jiang L., Tsao R. (2020). A comprehensive profiling of free, conjugated and bound phenolics and lipophilic antioxidants in red and green lentil processing by-products. Food Chem..

[29] He Y., Peng L., Xiong H., Liu W., Zhang H., Peng X., Zhu X., Guo F., Sun Y. (2023). The profiles of durian (*Durio zibethinus* Murr.) shell phenolics and their antioxidant effects on H_2_O_2_-treated HepG2 cells as well as the metabo-lites and organ distribution in rats. Food Res. Int..

[30] Vallverdú-Queralt A., Jáuregui O., Medina-Remón A., Andrés-Lacueva C., Lamuela-Raventós R.M. (2010). Improved characterization of tomato polyphenols using liquid chromatography/electrospray ionization linear ion trap quadrupole Orbitrap mass spectrometry and liquid chromatography/electrospray ionization tandem mass spectrometry. Rapid Commun. Mass Spectrom..

[31] Shen Z.H., Zhu C.X., Quan Y.S., Yang Z.Y., Wu S., Luo W.W., Tan B., Wang X.Y. (2018). Relationship between intesti-nal microbiota and ulcerative colitis: Mechanisms and clinical application of probiotics and fecal microbiota transplantation. World J. Gastroenterol..

[32] Machado A.P.D.F., Geraldi M.V., Do Nascimento R.D.P., Moya A.M.T.M., Vezza T., Diez-Echave P., Gálvez J.J., Cazarin C.B.B., Maróstica Júnior M.R. (2021). Polyphenols from food by-products: An alternative or complementary therapy to IBD conventional treatments. Food Res. Int..

[33] Chao L., Lin J., Zhou J., Du H., Chen X., Liu M., Qu Q., Lv W., Guo S. (2022). Polyphenol Rich Forsythia suspensa Ex-tract Alleviates DSS-Induced Ulcerative Colitis in Mice through the Nrf2-NLRP3 Pathway. Antioxidants.

[34] Nascimento R.D.P.D., Rizzato J.S., Polezi G., Moya A.M.T.M., Silva M.F., Machado A.P.D.F., Franchi Junior G.C., Borguini R.G., Santiago M.C.P.D., Paiotti A.P.R. (2023). Freeze-dried jaboticaba (*Myrciaria jaboticaba*) peel powder, a rich source of anthocyanins and phenolic acids, mitigates inflammation-driven colorectal cancer in mice. Food Bi-Osci..

[35] Xu B., Wang Y., Jiang L., Liu Z., Liu D., Zhao H., Li S., Wang X. (2023). Inhibitory effect of main phenolic acid compo-nents of *Jacobaea cannabifolia* (Less.) on inflammation caused by PM2.5. Front. Pharmacol..

[36] Li N., Li B., Zhang J., Liu X., Liu J., Li K., Pan T., Wang S., Diao Y. (2019). Protective effect of phenolic acids from Chebulae Fructus immaturus on carbon tetrachloride induced acute liver injury via suppressing oxidative stress, inflammation and apoptosis in mouse. Nat. Prod. Res..

[37] Ghasemi-Dehnoo M., Amini-Khoei H., Lorigooini Z., AnjomShoa M., Rafieian-Kopaei M. (2023). Ferulic acid ameliorates ulcerative colitis in a rat model via the inhibition of two LPS-TLR4-NF-κB and NF-κB-INOS-NO signaling pathways and thus alleviating the inflammatory, oxidative and apoptotic conditions in the colon tissue. Inflammopharmacology.

[38] Bains M., Kaur J., Akhtar A., Kuhad A., Sah S.P. (2022). Anti-inflammatory effects of ellagic acid and vanillic acid against quinolinic acid-induced rat model of Huntington’s disease by targeting IKK-NF-κB pathway. Eur. J. Pharmacol..

[39] Zhu L., Gu P., Shen H. (2019). Gallic acid improved inflammation via NF-κB pathway in TNBS-induced ulcerative colitis. Int. Immunopharmacol..

[40] Zielińska D., Zieliński H., Laparra-Llopis J.M., Szawara-Nowak D., Honke J., Giménez-Bastida J.A. (2021). Caffeic Acid Modulates Processes Associated with Intestinal Inflammation. Nutrients.

[41] Yang X., Sun X., Zhou F., Xiao S., Zhong L., Hu S., Zhou Z., Li L., Tan Y. (2023). Protocatechuic Acid Alleviates Dex-tran-Sulfate-Sodium-Induced Ulcerative Colitis in Mice via the Regulation of Intestinal Flora and Ferroptosis. Molecules.

[42] Seo S.H., Jo S., Truong T.T.M., Zhang G., Kim D., Lee M., Lee Y., Kang I. (2021). Peanut sprout rich in p-coumaric acid ameliorates obesity and lipopolysaccharide-induced inflammation and the inhibition of browning in adipocytes via mitochondrial activation. Food Funct..

[43] Maurer L.H., Cazarin C.B.B., Quatrin A., Minuzzi N.M., Costa E.L., Morari J., Velloso L.A., Leal R.F., Rodrigues E., Bochi V.C. (2019). Grape peel powder promotes intestinal barrier homeostasis in acute TNBS-colitis: A major role for dietary fiber and fiber-bound polyphenols. Food Res. Int..

[44] Ferguson L. (2014). Dietary interactions with the bacterial sensing machinery in the intestine: The plant polyphenol case. Front. Genet..

[45] Li Q., Liang X., Guo N., Hu L., Prasad M.E., Wu Y., Xue X., Wu L., Wang K. (2019). Protective effects of Bee pollen extract on the Caco-2 intestinal barrier dysfunctions induced by dextran sulfate sodium. Biomed. Pharmacother..

[46] Pastorelli L., De Salvo C., Mercado J., Vecchi M., Pizarro T. (2013). Central Role of the Gut Epithelial Barrier in the Pathogenesis of Chronic Intestinal Inflammation: Lessons Learned from Animal Models and Human Genetics. Front. Immunol..

[47] Antoni L., Nuding S., Wehkamp J., Stange E.F. (2014). Intestinal barrier in inflammatory bowel disease. World J. Gastroenterol..

[48] Gao R., Shen Y., Shu W., Jin W., Bai F., Wang J., Zhang Y., El-Seedi H., Sun Q., Yuan L. (2020). Sturgeon hydrolysates alleviate DSS-induced colon colitis in mice by modulating NF-κB, MAPK, and microbiota composition. Food Funct..

[49] Leppkes M., Neurath M.F. (2020). Cytokines in inflammatory bowel diseases—Update 2020. Pharmacol. Res..

[50] Li B., Alli R., Vogel P., Geiger T.L. (2014). IL-10 modulates DSS-induced colitis through a macrophage–ROS–NO axis. Mucosal Immunol..

[51] Li X., Mo K., Tian G., Zhou J., Gong J., Li L., Huang X. (2023). Shikimic Acid Regulates the NF-κB/MAPK Signaling Pathway and Gut Microbiota to Ameliorate DSS-Induced Ulcerative Colitis. J. Agric. Food Chem..

[52] Hayden M.S., Ghosh S. (2011). NF-κB in immunobiology. Cell Res..

[53] Hu H., Li Z., Zhu X., Lin R., Peng J., Tao J., Chen L. (2013). GuaLou GuiZhi decoction inhibits LPS-induced microglial cell motility through the MAPK signaling pathway. Int. J. Mol. Med..

[54] Espelin C.W., Goldsipe A., Sorger P.K., Lauffenburger D.A., de Graaf D., Hendriks B.S. (2010). Elevated GM-CSF and IL-1β levels compromise the ability of p38 MAPK inhibitors to modulate TNF-α levels in the human monocyt-ic/macrophage U937 cell line. Mol. Biosyst..

[55] Li H., Christman L.M., Li R., Gu L. (2020). Synergic interactions between polyphenols and gut microbiota in mitigating inflammatory bowel diseases. Food Funct..

[56] Parada Venegas D., De la Fuente M.K., Landskron G., González M.J., Quera R., Dijkstra G., Harmsen H.J.M., Faber K.N., Hermoso M.A. (2019). Short Chain Fatty Acids (SCFAs)-Mediated Gut Epithelial and Immune Regulation and Its Relevance for Inflammatory Bowel Diseases. Front. Immunol..

[57] Sun H., Chen Y., Cheng M., Zhang X., Zheng X., Zhang Z. (2018). The modulatory effect of polyphenols from green tea, oolong tea and black tea on human intestinal microbiota in vitro. J. Food Sci. Technol..

[58] Anhe F.F., Pilon G., Roy D., Desjardins Y., Levy E., Marette A. (2016). Triggering Akkermansia with dietary polyphenols: A new weapon to combat the metabolic syndrome?. Gut Microbes.

[59] Niu X., Shang H., Chen S., Chen R., Huang J., Miao Y., Cui W., Wang H., Sha Z., Peng D. (2021). Effects of *Pinus massoniana* pollen polysaccharides on intestinal microenvironment and colitis in mice. Food Funct..

[60] Cheng W., Lam K., Li X., Kong A.P., Cheung P.C. (2021). Circadian disruption-induced metabolic syndrome in mice is ameliorated by oat β-glucan mediated by gut microbiota. Carbohydr. Polym..

[61] Stojanov S., Berlec A., Štrukelj B. (2020). The Influence of Probiotics on the Firmicutes/Bacteroidetes Ratio in the Treatment of Obesity and Inflammatory Bowel disease. Microorganisms.

[62] Di Lorenzo F., De Castro C., Silipo A., Molinaro A. (2019). Lipopolysaccharide structures of Gram-negative populations in the gut microbiota and effects on host interactions. Fems Microbiol. Rev..

[63] Shi J., Xie Q., Yue Y., Chen Q., Zhao L., Evivie S.E., Li B., Huo G. (2021). Gut microbiota modulation and anti-nflammatory properties of mixed lactobacilli in dextran sodium sulfate-induced colitis in mice. Food Funct..

[64] Tian X., Yu Z., Feng P., Ye Z., Li R., Liu J., Hu J., Kakade A., Liu P., Li X. (2019). Lactobacillus plantarum TW1-1 Alleviates Diethylhexylphthalate-Induced Testicular Damage in Mice by Modulating Gut Microbiota and Decreasing Inflammation. Front. Cell. Infect. Microbiol..

[65] Le B., Yang S.H. (2018). Efficacy of *Lactobacillus plantarum* in prevention of inflammatory bowel disease. Toxicol. Rep..

[66] Guo H., Yu L., Tian F., Chen W., Zhai Q. (2023). The Potential Therapeutic Role of *Lactobacillaceae rhamnosus* for Treatment of Inflammatory Bowel Disease. Foods.

[67] Kim W., Han D.H., Jang Y.J., Park S., Jang S.J., Lee G., Han H.S., Ko G. (2021). Alleviation of DSS-induced colitis via *Lactobacillus acidophilus* treatment in mice. Food Funct..

