# Supplementary material for: Wheat Bran Polyphenols Ameliorate DSS-Induced Ulcerative Colitis in Mice by Suppressing MAPK/NF-κB Inflammasome Pathways and Regulating Intestinal Microbiota
Source: Foods. 2024 Jan 10;13(2):225. doi: 10.3390/foods13020225 (PMC10814686; doi:10.3390/foods13020225)
Supplement: Supplementary file 1 [file foods-13-00225-s001.zip › foods-2741256-supplementary.pdf]

Supplementary materials

Figure S1. MS/MS spectra and fragmentation patterns of selected phenolic components.

Table S1. Disease activity index (DAI) scoring criteria.

Table S2. Primer sequence for quantitative PCR.

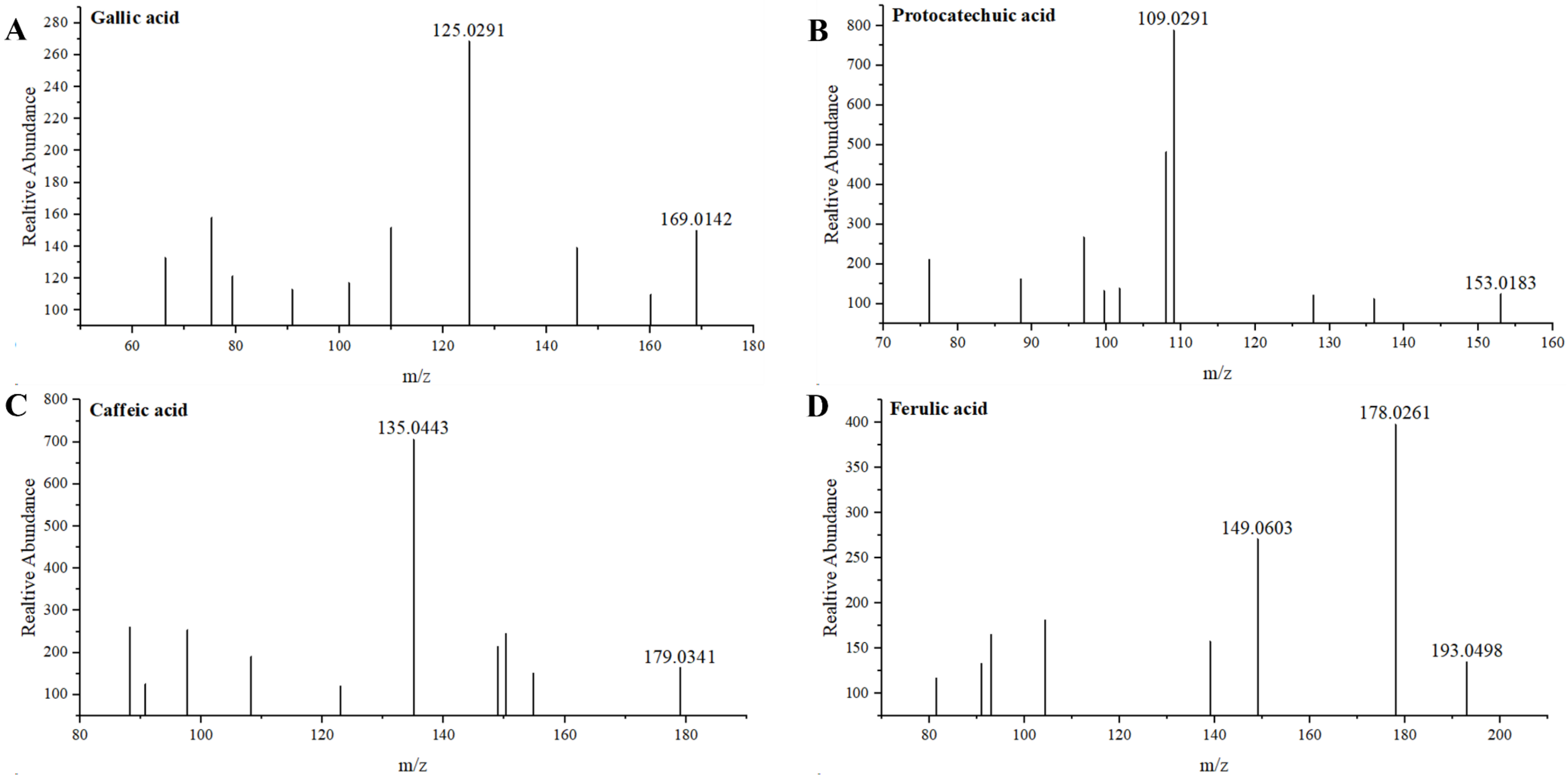

Figure S1. MS/MS spectra and fragmentation patterns of selected phenolic components. (A) Gallic acid; (B) Protocatechuic acid; (C) Caffeic acid; (D) Ferulic acid.

Table S1. Disease activity index (DAI) scoring criteria.

Body weight loss (%)	Stool consistency	Retal bleeding	Activity states	Score
No change	Normal	No observable blood	Normal	0
1-5	Loose stool	Small amount of blood in some stool	Some signs of poor condition	1
5-10	Mild diarrhea	Blood in stool regularly seen	Moderately poor condition	2
⩾10	Diarrhea	Blood in all stool	Bad condition	3

Table S2. Primer sequence for quantitative PCR.

Gene	Primer	Sequence (5'-3')
β-actin	Forward	ATGGAGCCGGACAGAAAAGC
(Mouse)	Reverse	TGGGAGGTGTCAACATCTTCTT
ZO-1	Forward	CCAGAGCCTCAGAAACCTCA
(Mouse)	Reverse	GCAGGAAGATGTGCAGAAGG
Occludin	Forward	GCGGAAAGAGTTGACAGTCC
(Mouse)	Reverse	TGCCTGAAGTCATCCACACT
Claudin-1	Forward	ACGGTCTTTGCACTTTGGTC
(Mouse)	Reverse	GGGAGAGGAGAAGCACAGTT
IκB-α	Forward	TGAAGGACGAGGAGTACGAGC
(Mouse)	Reverse	TGCAGGAACGAGTCTCCGT
NF-κB p65	Forward	TGTTCCAAAGAGCACCCAGG
(Mouse)	Reverse	TGCCAGGTCTGTGAACACTC
ERK	Forward	GGTTGTTCCCAAATGCTGACT
(Mouse)	Reverse	CAACTTCAATCCTCTTGTGAGGG
JNK	Forward	GTGGAATCAAGCACCTTCACT
(Mouse)	Reverse	TCCTCGCCAGTCCAAAATCAA
MAPK p38	Forward	TGACCCTTATGACCAGTCCTTT
(Mouse)	Reverse	GTCAGGCTCTTCCACTCATCTAT
